# Stereotactic core ablative radiation therapy for small hypoxic tumors: impact of dosimetric approaches and consequent optimization strategy in the context of spatially fractionated radiation therapy

**DOI:** 10.3389/fonc.2025.1568959

**Published:** 2025-09-30

**Authors:** Zhengzheng Xu, Salim Balik, Kaley Woods, Andrew Lim, Jason C. Ye, Eric L. Chang, Kristopher Lyons, Lijun Ma, Zhilei Shen, Lauren Lukas, Hualin Zhang

**Affiliations:** ^1^ Keck School of Medicine, University of Southern California, Los Angeles, CA, United States; ^2^ Norris Comprehensive Cancer Center, Keck School of Medicine, University of Southern California, Los Angeles, CA, United States; ^3^ Department of Radiation Oncology, University Hospitals Seidman Cancer Center, Cleveland, OH, United States

**Keywords:** stereotactic core ablative radiation therapy, treatment planning, spatially fractionated radiation therapy, lattice therapy, small target, VMAT

## Abstract

**Purpose:**

Stereotactic core ablative radiation therapy (SCART) delivers a single ablative dose core to the central hypoxic part while keeping low doses to the periphery of the tumor. This study evaluated the dosimetric impacts of various SCART planning approaches for small targets in the context of spatially fractionated radiation therapy (SFRT).

**Methods and materials:**

Using an anthropomorphic phantom, SCART plans were generated for cases with one spherical target, two spherical targets, one spherical target and one irregularly shaped target, and four spherical targets. All the spherical targets were 3 cm in diameter. One-third of the central gross target volume (GTV) was contoured as GTV_central to represent the hypoxic tumor volume, while the rest was contoured as GTV_peripheral for low-dose (3-Gy) coverage. Within each GTV, a small sphere with a diameter ranging from 0.5 to 1.5 cm was contoured at the center to represent the volume of a single high-dose core (V_SHDC). For the irregularly shaped target, both spherical (V_SHDC) and conformal (V_cSHDC) high-dose cores were used for comparisons. A single fraction of 15 Gy was prescribed to V_SHDC in all plans. Single- and dual-isocenter techniques were used for the case of two targets. Dosimetric parameters, which were usually used to describe SFRT plans, were compared for all SCART plans. The pros and cons of all planning approaches were elaborated.

**Results:**

The mean dose to V_SHDC was 17.0 ± 0.7 Gy. For multiple-target SCART plans, the peripheral GTV receiving less than 3 Gy (V_GTVp<3Gy_) ranged from 35.1% to 63.6%. No significant difference in dosimetric parameters was found between plans using a single isocenter and dual isocenters. For the irregularly shaped target, V_cSHDC improved the equivalent uniform dose (EUD) while the low-dose (3-Gy) coverage (V_GTVp<3Gy_) decreased. The average D10/D90 of all the plans was 8.0 ± 1.7. SCART used 1-cm-diameter V_SHDC (volume ratio of V_SHDC/GTV was within 2%–5%), demonstrating better dosimetric balance between high-dose coverage for GTV_central and low-dose coverage for GTV_peripheral.

**Conclusion:**

SCART for small targets is feasible; the plans demonstrated a comparable dosimetric quality as seen in the traditional SFRT plans for bulky tumors.

## Introduction

Lattice radiotherapy (LRT), which takes advantage of modern three-dimensional (3D) planning techniques, is an important development of spatially fractionated radiation therapy (SFRT) (1–3). LRT excels in flexibility and normal structure sparing capability over GRID therapy (4). By placing multiple high- and low-dose cores inside the target, the heterogeneous dose distribution of LRT aims to improve the radiation response and local tumor control probability for bulky (e.g., larger than 6-cm diameter) or radioresistant tumors without increasing the toxicity (5).

It is believed that one major benefit of SFRT is improving the systemic anti-tumor immunity through the non-targeted effects, such as the bystander or radiation-induced abscopal effects. The non-targeted effects depend on the relationship between the irradiated and non-irradiated cancer cells, as well as the proximity to the original treatment site (6–8). The non-irradiated area inside the target may create immune reservoirs that are prompted by those immune-stimulatory cytokines in the adjacent sites (9, 10). It is further stipulated that the radiation-induced abscopal or bystander effect is a regional radiation-induced systemic effect that extends outside the treated volume and can trigger the regression of the non-irradiated parts of the tumor (8, 9). Another effect called the cohort effect occurs favorably under heterogeneous irradiation. With the cohort effect, the high-dose-irradiated cells may affect low-dose-irradiated cells and vice versa, although early studies have indicated that the cohort effect may be limited to a distance of millimeters within the irradiated target (9, 11). Markovsky et al. reported their partial irradiation study with murine models and found that partial irradiation using a single dose of 10 Gy led to tumor responses similar to those of fully irradiated tumors in immunocompetent mice (12).

“Oxygen-guided radiotherapy” with SFRT, also known as stereotactic core ablative radiation therapy (SCART), is a new SFRT application that has not been fully investigated. Compared to a traditional stereotactic body radiation therapy (SBRT) plan in which an ablative dose is uniformly delivered to the whole target, in SCART, the central hypoxic part of the tumor is treated with an ablative dose, while the oxygen-rich periphery of the tumor receives a much lower dose (e.g., dose less than 3 Gy as seen in GRID therapy valley doses) for the purpose of achieving an intensive cell killing at the center and improving stimulations in the tumor microenvironment that allows the bystander, abscopal, cohort, and other immunological effects to occur (8, 13–15). The hypothesis is that anti-immunogenic cell death will be more significant when delivering high doses to the central hypoxic tumor segment while sparing the lymphocytes at the tumor periphery (13, 16) (17).

Massaccesi et al. investigated the feasibility of planning techniques for irradiating the hypoxic core of bulky tumors (>6-cm diameter) using volumetric modulated arc therapy (VMAT) (16). They also evaluated the dosimetric properties using different normalization methods. Yu et al. created a boost volume inside the target as a single high-dose core (50 Gy in five fractions) for unresectable bulky sarcomas (18). They reported a response rate of 88.9% and a mean tumor reduction of 49.5% without any Grade 3 or higher toxicities. A phase I clinical trial of SCART (21 patients) used a total dose of 15 to 24 Gy in one to three fractions and demonstrated significant tumor shrinkage after SCART, with no more than Grade 3 toxicity observed (19).

One major challenge of treating small tumors with SFRT is placing multiple high-dose cores inside the small targets while achieving satisfactory peak valley dose ratios as recommended by the Radiosurgery Society (RSS) SFRT working group white papers. Delivering very small high-dose cores may require beam apertures of less than 5 × 5 mm^2^, which requires a high-resolution multi-leaf collimator (MLC). Moreover, additional physics measurements are required to validate the accuracy of small-field dosimetry. Therefore, the single high-dose core technique, SCART, shines as a new way to achieve SFRT for small targets. This study aims to fill the knowledge gap of small-target SCART, including the dosimetric impact of different planning techniques with spherical and irregularly shaped small target volumes, different high-dose core volume sizes, and single or dual isocenters. All the dose metrics recommended by the RSS SFRT working group white papers were reported (20).

## Methods and materials

**Figure 1:**
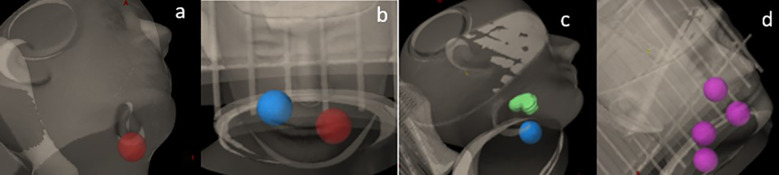
Sample cases with **(a)** one spherical target, **(b)** two spherical targets, **(c)** one spherical target and one irregularly shaped target, and **(d)** four spherical targets.

A Rando head phantom was scanned with a CT simulator using 120 kVp at 1-mm slice thickness. A series of SCART plans with one, two, and four targets were generated using Varian’s Eclipse (v15.6) treatment planning system (TPS). The plans were delivered using the TrueBeam STX with 120 HD MLC (Varian Inc., Palo Alto,CA, USA). The dosimetric parameters were then compared with the results from SCART plans reported by Massaccesi et al (16). As shown in **Figure 1**, sample cases included 1) one spherical target, 2) two spherical targets, 3) one spherical target and one irregularly shaped target, and 4) four spherical targets. Each spherical target was 3 cm in diameter (volume, 14.14 cm^3^). The irregularly shaped target was contoured with an effective volume of 14.1 cm^3^. There was a 6-cm center-to-center distance between two adjacent targets. However, in the sample case of four targets, two targets were deliberately placed with a center-to-center distance of 4 cm.

**Figure 2:**
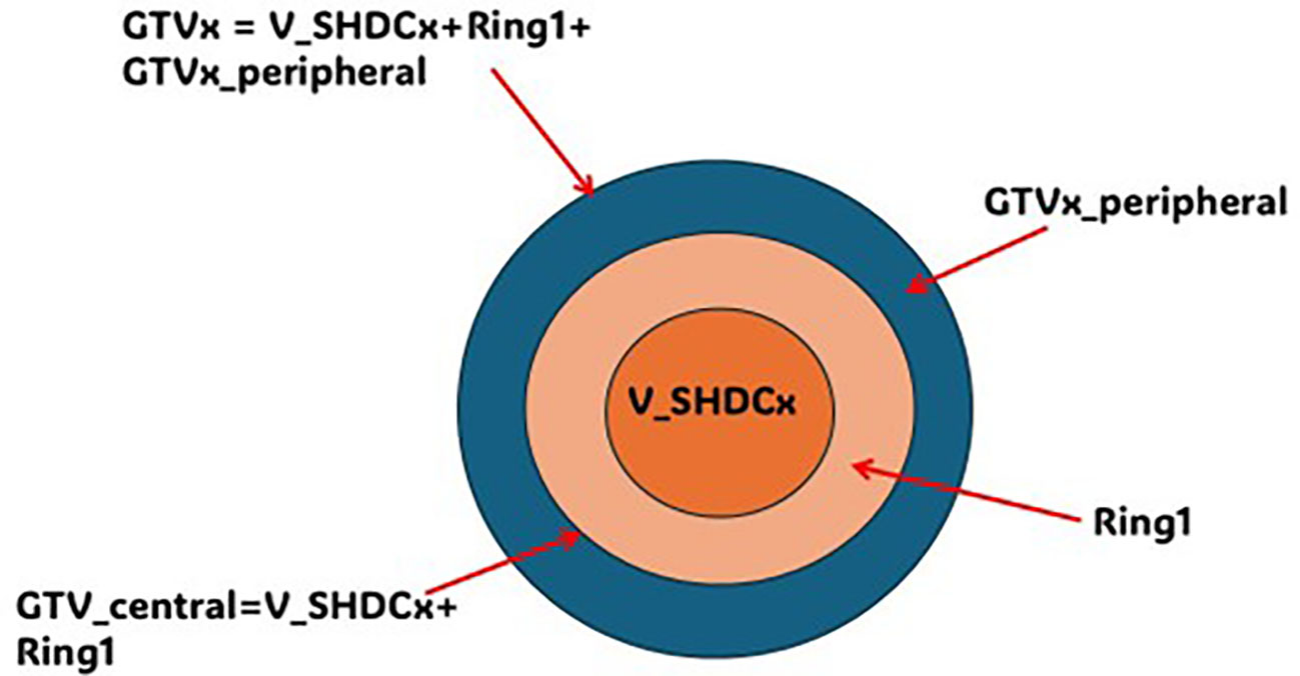
Illustration of GTVx, GTVx_peripheral, GTVx_central, Ring1, and V_SHDCx. x, target 1–4; GTVx_central = V_SHDCx + Ring1; GTVx = GTVx_central + GTVx_peripheral.


As shown in **Figure 2**, each target was named as gross target volume (GTVx), where x represented a specific target from one to four. A central contour V_SHDCx represented the volume of a single high-dose core. The contouring of V_SHDCx followed the method used in the study by Massaccesi et al. (16) Massaccesi et al. used the central one-third of the GTV as the high-dose core for planning, while we used V_SHDCx of 0.5, 1.0, and 1.5 cm in diameters to investigate the dosimetry impact of V_SHDCx volume sizes. To achieve better dose conformity, additional planning contours, such as the ring structure, were added in the plan optimization. The central one-third of the GTVx volume (diameter of 2.1 cm) was contoured as GTVx_central to represent the central hypoxic tumor zone, and the rest of GTVx outside GTVx_central was contoured as GTVx_peripheral (16). Typically, the ratio of the central high-dose core volume to the GTV is defined by Equation 1.

(1) $$R_{SCART}=\frac{V\_SHDC}{V\_GTV}$$

where V_SHDC is the volume of the high-dose core(s) and V_GTV is the volume of the GTV. Therefore, with the more equivalent dimension of _V_SHDC_, L_V_SHDC_ can be determined using Equation 2:


(2) $$L_{V\_SHDC}=f\times R_{SCART}\times L_{GTV}$$

where f is the dimension conversion factor for volume or diameter. $L_{V\_SHDC}$ and L_GTV_ are the dimensions (e.g., volume or diameter) of V_SHDC and GTV, respectively.

The volume between V_SHDCx and GTVx_central was contoured as Ring1, which was used to control the dose falloff outside the high-dose core (V_SHDCx).

**Figure 3:**
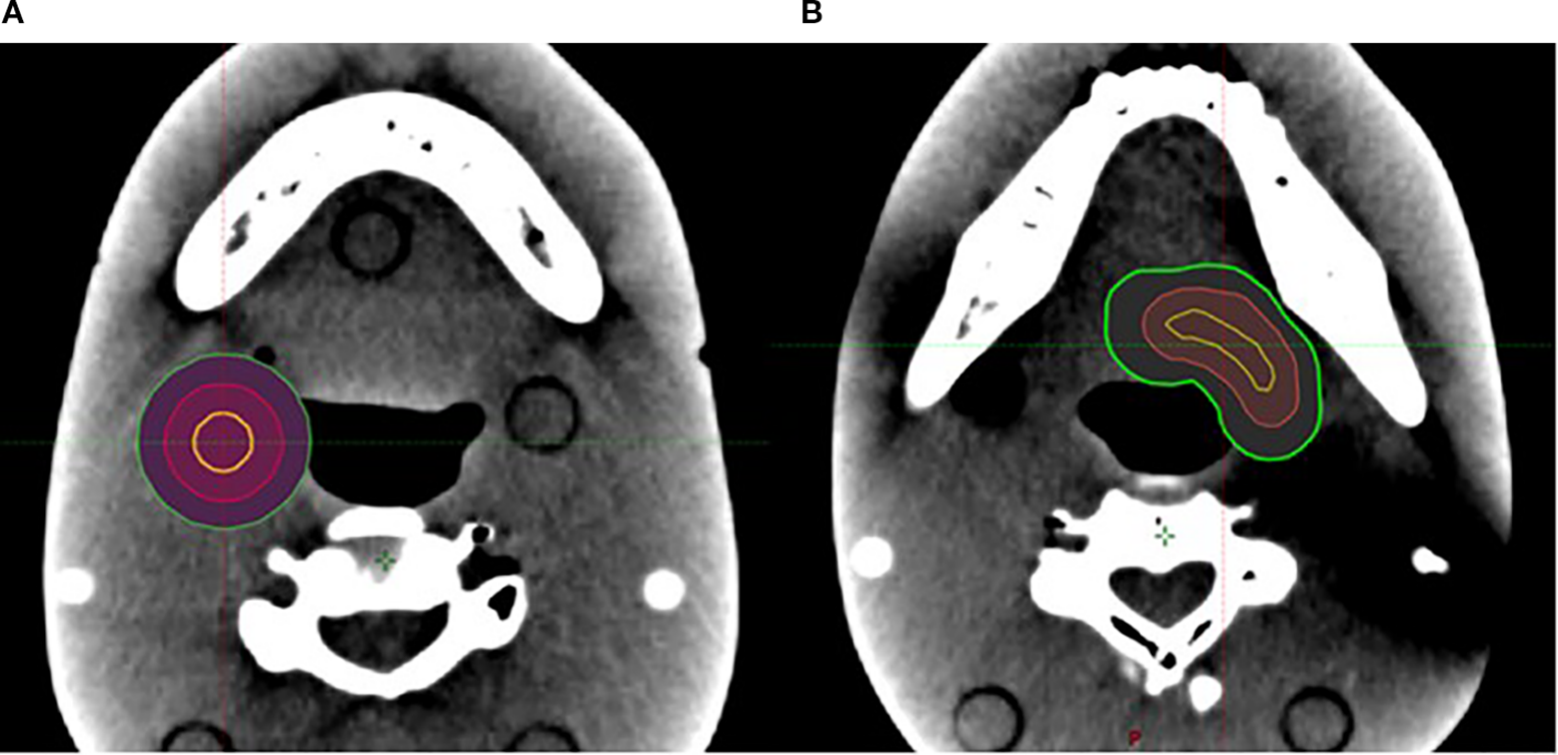
**(A)** One 1-cm-diameter spherical V_SHDCx (yellow) was placed in the middle of a spherical target. **(B)** One V_cSHDCx in yellow was placed in the irregularly shaped target (volume equivalent to the spherical V_SHDCx of 1-cm diameter). Red, GTVx_central; green, GTVx.

The VMAT plan used a 2-mm calculation resolution. For cases with one target, the SCART plan used two arcs, and the isocenter was placed at the center of the target. For cases with multiple targets, the isocenters of SCART plans that used a single isocenter were placed at the geometric center of all the targets, while the isocenters of dual-isocenter plans were placed at the center of each target (the dual-isocenter technique was used for cases of two targets only). For the irregularly shaped targets, a conformal V_SHDCx (i.e., V_cSHDCx) contour with its margin subtracted from the irregularly shaped target was also used for planning. The volume of V_cSHDCx was equivalent to the volume of spherical V_SHDCx of 1-cm diameter (**Figure 3**). Dosimetric comparisons were performed for plans using V_cSHDCx and V_SHDCx. **Table 1** demonstrates the detailed dose constraints used for SCART optimization.

**Table 1:** Dose constraints used for target sub-structures in SCART plan optimization.

Structure constraints	Volume %	Dose (gy)	Priority
V_SHDCx/V_cSHDCx			
Upper	<10	18.75	60
Lower	100	14.25	100
Ring1			
Upper	<10	9.50	50
GTVx_peripheral			
Upper	≥40	3.00	80

SCART, stereotactic core ablative radiation therapy.

For the sample cases with two targets, the dose metrics of plans generated using either single or dual isocenters were compared. The plan with four targets just used a single isocenter per our clinical protocol. The RSS SFRT white papers recommend dosimetric parameters (20), including D5, D10, D50, D90, and D95 (doses covering 5%, 10%, 50%, 90%, and 95% of the target volume, respectively), D5/D95, D10/D90, and equivalent uniform dose (EUD) to be reported in each SFRT plan. We also reported the maximal dose (Dmax) of GTVx and the percentage volume of GTVx_peripheral receiving less than 3 Gy (V_GTVp<3Gy_). In the EUD calculations, we assumed the GTVx as semi-radiosensitive cancer cells where the α/β ratio was 10 Gy and the survival fraction (SF) in a 2-Gy uniform dose field was 0.5 (α = 0.289 Gy^−1^, β = 0.0289 Gy^−2^, and 1-hour repair half-time) (21, 22).

The treatment plan delivery quality was experimentally verified. An electronic portal imaging device (EPID)-based two-dimensional dosimetric quality assurance (QA) system was employed to check all SCART plans. The passing rate threshold of 95% using 3% and 2-mm gamma criteria was used.

## Results

**Figure 4:**
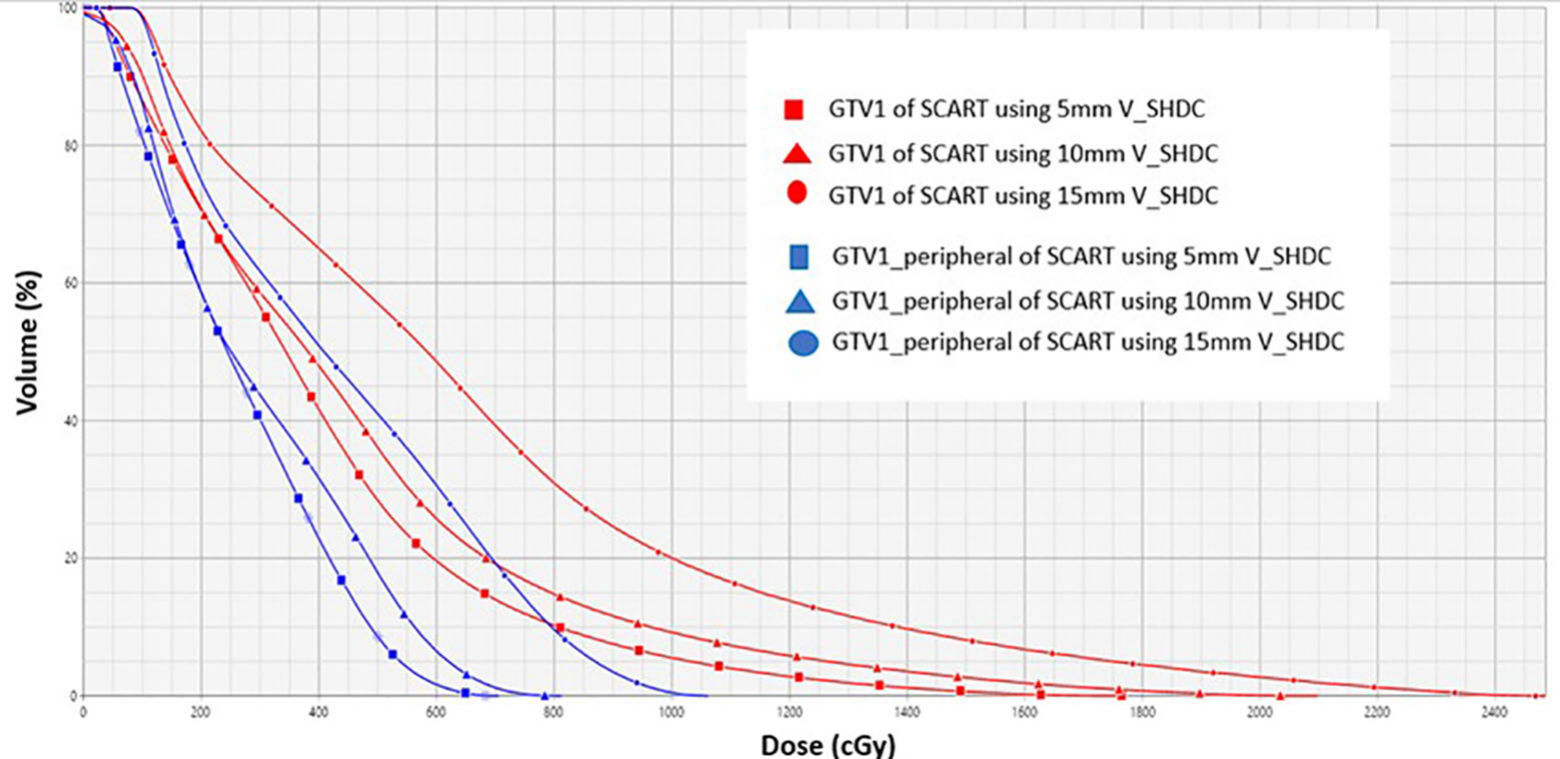
DVH curves of GTV1 (red) and GTV1_peripheral (blue) from SCART plans for using 5-, 10-, and 15-mm diameters of V_SHDC. SCART, stereotactic core ablative radiation therapy.

**Figure 4** compares the dose volume histogram (DVH) curves (e.g., GTV1 and GTV1_peripheral) of SCART plans of a single spherical target using different sizes of V_SHDC. **Table 2** summarizes the dose metrics of all the SCART plans. For all the SCART plans, the mean dose of V_SHDCx was 17.0 ± 0.7 Gy, and the average V_GTVp<3Gy_ was 49.7% ± 9.4%. The SFRT dose metrics of average D10/D90, D5/D95, and EUD of all the GTVx were 8.0 ± 1.7, 12.8 ± 3.3, and 3.9 ± 0.8 Gy, respectively. The mean D10/D90 of SCART plans was higher than the typical D10/D90 (e.g., 3–6) of SFRT plans for bulky targets (23–28).

**Table 2:** Summary of DVH parameters for SCART plans.

SCART plans	V_SHDC	GTVx*									GTV_peripheral*
	Dmean (gy)	D5 (gy)	D10 (gy)	D50 (gy)	D90 (gy)	D95 (gy)	Dmax (gy)	EUD (gy)	D10/D90	D5/D95	V_GTVp<3Gy_
1. One spherical target											
1.1. Single isocenter											
1.1.1. V_SHDC (d = 0.5 cm)	16.4	10.9	8.4	3.4	0.9	0.7	18.3	3.1	9.3	15.6	53.2
1.1.2. V_SHDC (d = 1.0 cm)	17.1	12.7	9.6	3.8	1.2	0.9	21.0	3.4	8.0	14.1	54.1
1.1.3. V_SHDC (d = 1.5 cm)	17.2	17.0	15.1	7.0	2.0	1.7	21.6	5.2	7.6	10.0	30.5
Mean ± SD	16.9 ± 0.4	13.5 ± 2.6	11.0 ± 2.9	4.7 ± 1.6	1.4 ± 0.5	1.1 ± 0.4	20.3 ± 1.4	3.9 ± 0.9	8.3 ± 0.7	13.2 ± 2.4	45.9 ± 10.9
2. Two spherical targets											
2.1. Single isocenter											
2.1.1. V_SHDC (d = 0.5 cm)	15.8	11.2	8.7	3.7	0.9	0.7	18.5	3.2	9.7	16.0	54.8
2.1.2. V_SHDC (d = 1.0 cm)	17.0	12.8	9.7	3.8	1.1	0.9	20.6	3.5	8.8	14.5	54.6
2.1.3. V_SHDC (d = 1.5 cm)	16.6	17.1	15.3	6.9	2.2	1.9	21.5	5.0	7.0	9.0	30.2
Mean ± SD	16.5 ± 0.5	13.7 ± 2.5	11.2 ± 2.9	4.8 ± 1.5	1.4 ± 0.6	1.2 ± 0.5	20.2 ± 1.3	3.9 ± 0.8	8.5 ± 1.1	13.2 ± 3.0	46.5 ± 11.5
2.2. Dual isocenters (d = 1.0 cm)	16.8	13.2	10.2	3.8	0.8	0.6	20.2	3.3	12.8	22.0	54.5
3. One spherical target and one irregularly shaped target											
3.1. Single isocenter											
3.1.1. V_SHDC (d = 0.5 cm)	16.8	11.7	8.1	2.7	1.1	0.8	19.1	3.0	7.4	14.6	63.6
3.1.2. V_SHDC (d = 1.0 cm)	17.5	13.9	10.5	3.8	1.6	1.2	20.7	3.6	6.6	11.6	52.5
3.1.3. V_SHDC (d = 1.5 cm)	17.3	17.3	14.7	5.2	2.4	1.5	20.8	4.1	6.1	11.5	41.5
3.1.4. V_cSHDC (d = 0.5-cm equiv.)	16.6	13.4	10.8	4.3	1.4	1.2	21.7	3.4	8.4	11.2	56.3
3.1.5. V_cSHDC (d = 1.0-cm equiv.)	15.8	16.2	15.1	8.8	1.8	1.5	20.2	3.9	7.7	10.8	49.8
3.1.6. V_cSHDC (d = 1.5-cm equiv.)	16.9	17.2	15.9	5.8	2.2	1.7	20.0	4.7	7.2	10.1	41.1
Mean ± SD	16.8 ± 0.5	15.0 ± 2.1	12.5 ± 2.9	5.1 ± 1.9	1.8 ± 0.4	1.3 ± 0.3	20.4 ± 0.8	3.8 ± 0.5	7.2 ± 0.7	11.6 ± 1.4	50.8 ± 7.9
3.2. Dual isocenters (d = 1.0 cm)	17.2	13.2	10.3	3.8	1.0	0.8	20.2	4.4	10.3	16.5	54.0
4. Four spherical targets											
4.1. Single isocenter											
4.1.1. V_SHDC (d = 0.5 cm)	17.8	10.3	7.8	3.3	1.3	1.0	18.2	3.3	6.5	10.3	53.8
4.1.2. V_SHDC (d = 1.0 cm)	18.3	13.1	10.5	4.3	1.7	1.3	22.5	3.8	6.2	10.1	53.2
4.1.3. V_SHDC (d = 1.5 cm)	18.1	17.0	14.5	5.9	2.2	1.9	21.4	5.5	6.1	8.9	35.1
Mean ± SD	18.1 ± 0.2	13.5 ± 2.7	10.9 ± 2.8	4.5 ± 1.1	1.7 ± 0.4	1.4 ± 0.4	20.7 ± 1.8	4.2 ± 0.9	6.3 ± 0.2	9.8 ± 0.6	47.4 ± 8.7

SD, standard deviation; SCART, stereotactic core ablative radiation therapy.

*For the case of multiple targets, the dose metrics were averaged for all GTVx or GTVx_peripheral. d = 1.0 cm means the diameter is 1 cm.

**Figure 5:**
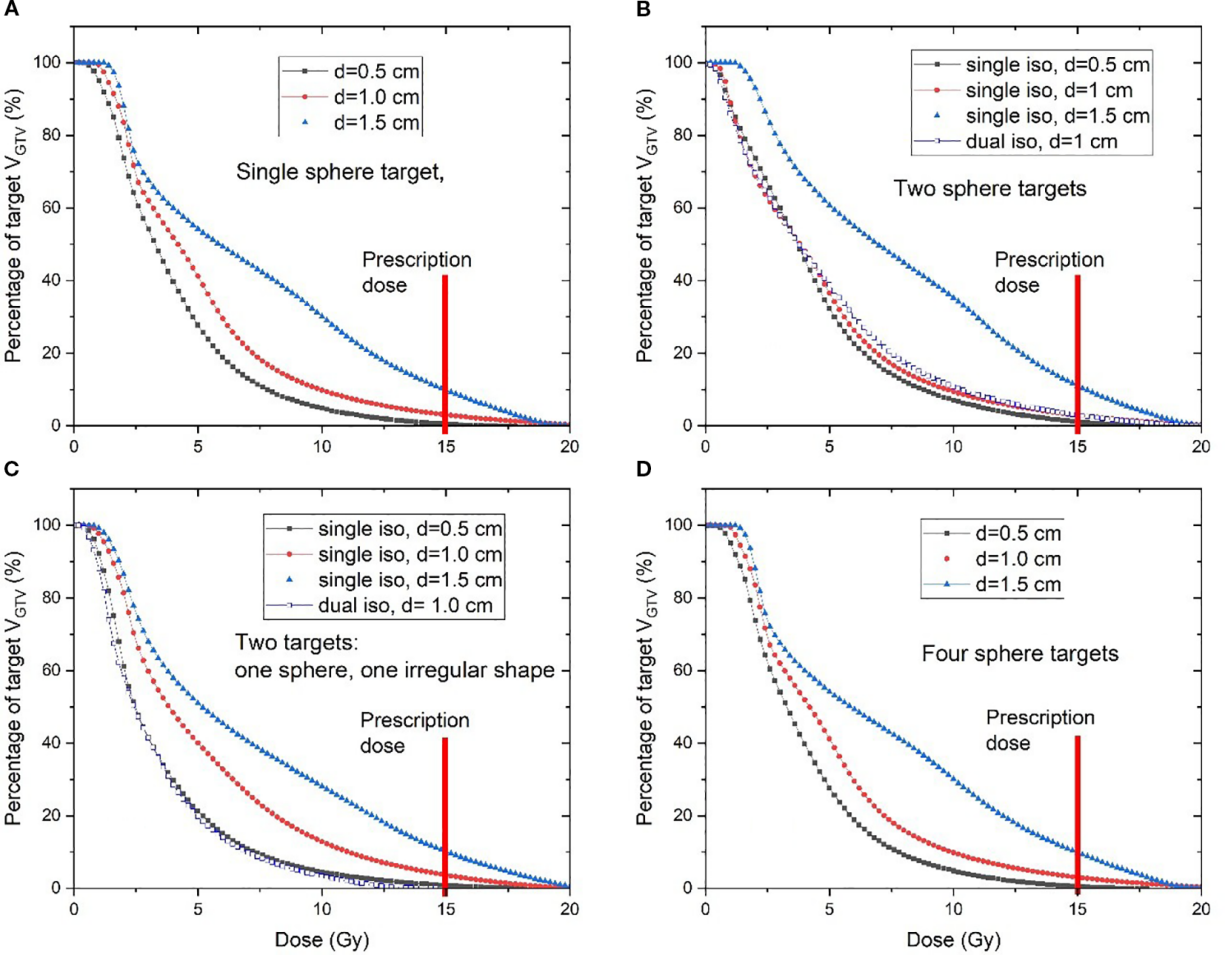
DVH curves of four different clinical scenarios: **(A)** single spherical target, **(B)** two spherical targets, **(C)** one spherical target and one irregularly shaped target, and **(D)** four spherical targets. Various diameters of V_SHDCx were used in each scenario. The SCART prescription dose is marked by the red line. SCART, stereotactic core ablative radiation therapy.

The plans that used V_SHDCx of 1.0-cm diameter demonstrated high V_GTVp<3Gy_ (52.9% ± 0.7%) and EUD (3.7 ± 0.3 Gy). Plans that used 0.5-cm-diameter V_SHDCx demonstrated the highest mean V_GTVp<3Gy_ (57.1% ± 4.6%), D10/D90 (8.5 ± 0.9), and D5/D95 (14.7 ± 1.0), with lower EUD (3.1 ± 0.1 Gy). In comparison, those that used 1.5-cm-diameter V_SHDCx demonstrated the lowest V_GTVp<3Gy_ (35.6% ± 4.7%), D10/D90 (6.8 ± 0.7), and D5/D95 (10.0 ± 0.9) and the highest EUD (4.9 ± 0.5 Gy), indicating less dose heterogeneity but higher GTVx dose coverage. As shown in **Figure 5** and **Table 2**, when using V_SHDCx of the same size, the dose metrics of GTVx were less affected by factors such as the total number of targets and isocenters. Compared to the plans using 0.5- and 1.0-cm V_SHDCx, those using 1.5-cm-diameter V_SHDCx had significantly higher dose coverage of GTVx.

The QA of all the plans passed the gamma analysis (95% passing rate using 3% and 2-mm gamma criteria). There was no significant difference between plans using a single isocenter and dual isocenters. Compared to the plans of irregularly shaped targets using spherical V_SHDCx, those using conformal high-dose cores (V_cSHDCx) demonstrated higher EUD (4.0 ± 0.5 *vs*. 3.6 ± 0.4 Gy) while decreased V_GTVp<3Gy_ (49.1 ± 6.2 *vs*. 52.5 ± 9.0).

## Discussion

Per the report, Massaccesi et al. delivered 10 Gy to the central one-third of the GTV (high-dose core) and optimized the volume of less than 2 Gy in the periphery of GTV (V_GTVp<2Gy_). They reported that, in the SFRT for lesions of less than 6-cm diameter, their V_<2Gy_ ranged from 42.9% to 48.4% with approximately 130% hotspot in the central GTV (16). The SCART plans generated in this study all achieved V_GTVp<3Gy_ of at least 30%. As shown in **Table 2**, in our study, when using a 1.0-cm-diameter V_SHDC, the single target SCART plan demonstrated a higher percentage of low-dose volume inside GTV_peripheral compared to the one reported by Massaccesi et al. (16) (54.1% *vs*. 42.9%). The dose indexes of SCART plans demonstrated comparable dose inhomogeneity inside the target compared to the typical lattice VMAT plans (24, 28–32), although the proton GRID plans demonstrated much higher D10/D90 of 19.8 (23). SFRT plans made by Rivera et al. (15) reported a mean peak valley dose ratio (PVDR) of 13.3 using a 0.3-mm-wide planar mini-beam array.

As for three-dimensional lattice SFRT plans that involve multiple vertices, a proper size selection and distribution of vertices will have a significant dosimetric impact (33). Grams et al. reported that their lattice VMAT plans for large tumors (median volume of 301 cm^3^) using vertices of 1-cm diameter (2-cm center-to-center separation) demonstrated the highest EUD compared to plans with vertices of 1.5-cm diameter (3-cm center-to-center separation) (23). Several studies have discussed ideas to develop an automated lattice planning technique that can help optimize the size, location, and separation of the high-dose vertices (29, 30, 32). The vertices of 1- to 2-cm diameters are commonly adopted in different studies, as the size in this range enabled sufficient dose coverage and flexibility of vertex placement (1, 14, 23, 28–30, 32, 34, 35).

For an SFRT plan, a well-designed and sufficient low-dose zone (valley dose area) is crucial not only for keeping a low level of toxicity but also for stimulating immunomodulation effects (9, 26). To maintain a sufficient volume receiving 3 Gy or less in the periphery of the GTV while delivering an ablative dose to the central core in such a small target volume, the plan must have a sharp dose falloff outside the GTV center. However, sufficient dose coverage for GTV_central is also essential for tumor control. In this study, V_SHDCx of 0.5-cm diameter demonstrated better low-dose delivery in the periphery of GTV (V_GTVp<3Gy_), indicating potentially better immune preservation. However, a very small V_SHDCx cannot provide sufficient cancer cell killing inside the target. V_SHDCx of 1-cm diameter demonstrated better balance between high-dose coverage for GTV_central and low-dose control in GTV_peripheral. Plans using 1.5-cm-diameter V_SHDCx demonstrated better GTV coverage but at the expense of higher dose in the peripheral GTV and, as such, may lose the benefits of heterogeneous dose delivery attempting to spare the tumor microenvironment. A clinically verified effective size of V_SHDC can further leverage this technique.

In addition to the absolute size of V_SHDCx, we investigated the relationship between the total volume of the high-dose cores and tumor volume. Based on our search of lattice VMAT studies, the ratios of the total volume of vertices to the overall GTV ranged from 2% to 5% (1, 6, 36). In a study using photon beams for SCART, R_SCART_ of up to 11% was reported, while Li et al. reported R_SCART_ = 5% in the proton-based SCART study (19, 37). Another approach for defining the size of V_SHDC is being evaluated, which involves using a ratio between the peripheral and prescription doses instead of a volume ratio. Currently, there is no consensus on the optimal size or ratio for V_SHDC in SCART planning. There are several studies that have reported the ratios, while more clinical data are needed to standardize V_SHDC contouring for planning. Jin et al. used 36 high-dose spheres of 0.5-cm diameter for a GTV of 112.9 cm^3^, which corresponds to a ratio of 2.1% for the total volume of V_SHDC to the total volume of GTV. They used a non-coplanar VMAT planning technique and reported a PVDR of 2 to 2.5 (38). Massaccesi et al. used the central one-third of the GTV as the high-dose core, which corresponds to a ratio of 3.0% (16). Yang et al. reported a ratio of 10.6% when using 15 Gy for V_SHDC and a ratio of 4.5% when the dose was escalated to 21 Gy (19).

In this study, the average ratios of the total volume of V_SHDCx to the total volume of GTVx for diameters of 0.5, 1.0, and 1.5 cm were 0.5%, 3.6%, and 12.5%, respectively. This is consistent with the lattice therapy criteria (1, 39). The V_SHDC/GTV ratio for a 1-cm high-dose core was in the range of 2% to 5%, which supports the conclusion that SCART plans of 1 cm V_SHDCx can generate a more dosimetrically balanced plan for the targets generated in this study. However, when using SCART to treat tumors of various sizes, other high-dose core sizes may also be considered to achieve specific dosimetric goals for desired clinical outcomes.

**Figure 6:**
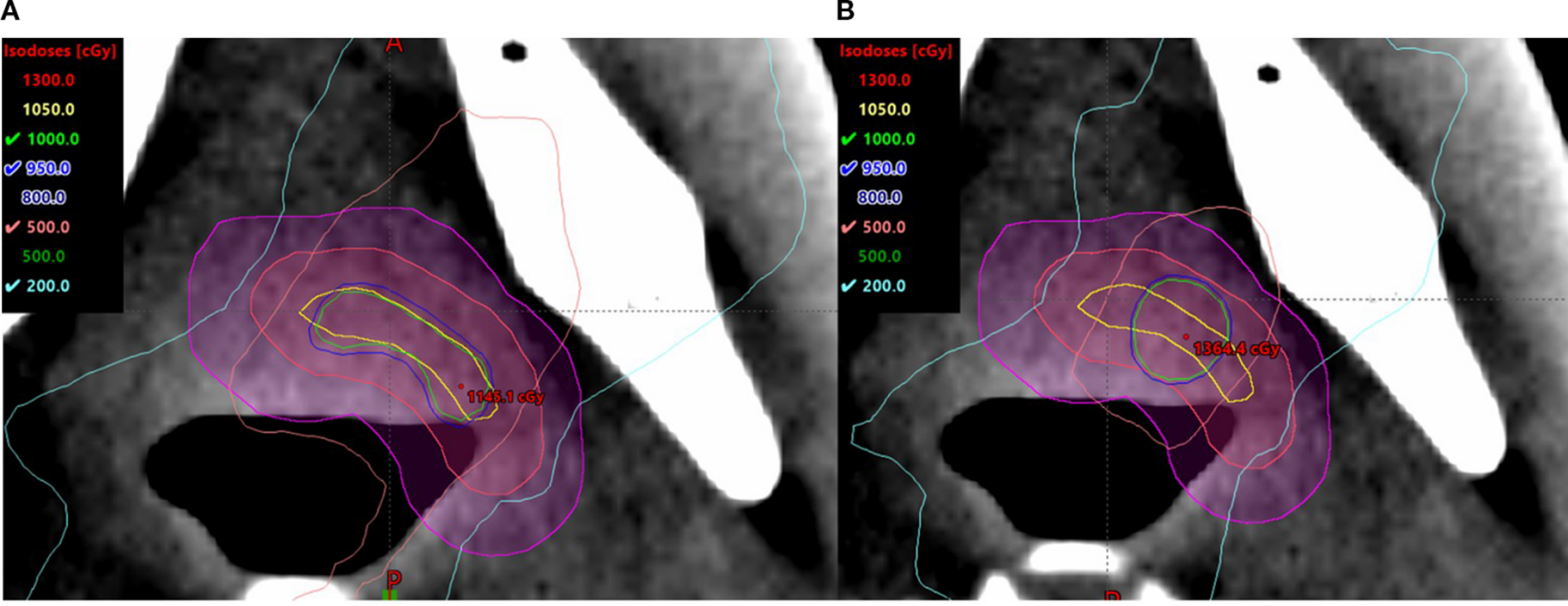
Irregularly shaped target using V_cSHDC **(A)** and spherical V_SHDC of an equivalent volume **(B)**.

The small, irregularly shaped targets are found to be prone to being affected by the size and location of the high-dose cores. Compared to the spherical V_SHDCx, the plans with irregularly shaped targets demonstrated greater EUDs (**Table 2**) when using a conformal structure (V_cSHDCx). However, the plans using conformal high-dose core structures required more dose modulation, resulting in approximately 30% more monitor units compared to the plans using spherical high-dose cores. This means that higher EUDs were delivered at the cost of a 30% longer running time. As shown in **Figure 6**, there was more dose spillage in the periphery and outside the GTV. Therefore, plans using spherical V_SHDC demonstrated better V_GTVp<3Gy_ compared to those using irregularly shaped V_cSHDC (**Table 2**).

One limitation of this study is that the plans were based on the anthropomorphic phantom instead of real patients; thus, the impact on surrounding normal tissues was not investigated. A future study is guaranteed to evaluate the impact of SCART on normal structures from retrospective patient data.

## Conclusion

SCART, or single high-dose core SFRT, is a feasible approach for treating single and multiple small targets with sufficient heterogeneous dose modulation. SCART plans for small targets can achieve a comparable dosimetric quality to SFRT plans that use multiple high-dose cores. The size of the high-dose core and the planning approach have a significant impact on dose metrics, which ultimately influence treatment outcomes. At this stage, the optimal high-dose core size and planning approach are expected to be determined by clinical goals and, eventually, clinical trial data.

## Data Availability

The original contributions presented in the study are included in the article/supplementary material. Further inquiries can be directed to the corresponding author.
